# Enzymatic chokepoints and synergistic drug targets in the sterol biosynthesis pathway of *Naegleria fowleri*

**DOI:** 10.1371/journal.ppat.1007245

**Published:** 2018-09-13

**Authors:** Wenxu Zhou, Anjan Debnath, Gareth Jennings, Hye Jee Hahn, Boden H. Vanderloop, Minu Chaudhuri, W. David Nes, Larissa M. Podust

**Affiliations:** 1 Department of Chemistry and Biochemistry, Texas Tech University, Lubbock, Texas, United States of America; 2 Center for Discovery and Innovation in Parasitic Diseases, Skaggs School of Pharmacy and Pharmaceutical Sciences, University of California San Diego, La Jolla, California, United States of America; 3 Department of Microbiology and Immunology, Meharry Medical College, Nashville, Tennessee, United States of America; University of Virginia, UNITED STATES

## Abstract

*Naegleria fowleri* is a free-living amoeba that can also act as an opportunistic pathogen causing severe brain infection, primary amebic meningoencephalitis (PAM), in humans. The high mortality rate of PAM (exceeding 97%) is attributed to (i) delayed diagnosis, (ii) lack of safe and effective anti-*N*. *fowleri* drugs, and (iii) difficulty of delivering drugs to the brain. Our work addresses identification of new molecular targets that may link anti-*Naegleria* drug discovery to the existing pharmacopeia of brain-penetrant drugs. Using inhibitors with known mechanism of action as molecular probes, we mapped the sterol biosynthesis pathway of *N*. *fowleri* by GC-MS analysis of metabolites. Based on this analysis, we chemically validated two enzymes downstream to CYP51, sterol C24-methyltransferase (SMT, ERG6) and sterol Δ^8^−Δ^7^ -isomerase (ERG2), as potential therapeutic drug targets in *N*. *fowleri*. The sterol biosynthetic cascade in *N*. *fowleri* displayed a mixture of canonical features peculiar to different domains of life: lower eukaryotes, plants and vertebrates. In addition to the cycloartenol→ergosterol biosynthetic route, a route leading to *de novo* cholesterol biosynthesis emerged. Isotopic labeling of the *de novo*-synthesized sterols by feeding *N*. *gruberi* trophozoites on the U^13^C-glucose-containing growth medium identified an exogenous origin of cholesterol, while 7-dehydrocholesterol (7DHC) had enriched ^13^C-content, suggesting a dual origin of this metabolite both from *de novo* biosynthesis and metabolism of scavenged cholesterol. Sterol homeostasis in *Naegleria* may be orchestrated over the course of its life-cycle by a “switch” between ergosterol and cholesterol biosynthesis. By demonstrating the growth inhibition and synergistic effects of the sterol biosynthesis inhibitors, we validated new, potentially druggable, molecular targets in *N*. *fowleri*. The similarity of the *Naegleria* sterol Δ^8^−Δ^7^ -isomerase to the human non-opioid σ_1_ receptor, implicated in human CNS conditions such as addiction, amnesia, pain and depression, provides an incentive to assess structurally diverse small-molecule brain-penetrant drugs targeting the human receptor for anti-*Naegleria* activity.

## Author summary

Sterols are important constituents of cell membranes. In a unicellurar organism, such as the human pathogen *N*. *fowleri*, the cell membrane delineates the physical boundaries of the cell and mediates interactions of the organism with its environment. *N*. *fowleri* is a free-living amoeba that may infect the human brain causing a fulminant infection called primary amebic meningoencephalitis (PAM). PAM has resulted in death in >97% of reported cases. Understanding the molecular and cellular biology of *N*. *fowleri* will facilitate the rational development of new therapeutic interventions. Using inhibitors targeting different enzymatic steps in the sterol biosynthesis pathway, we mapped metabolic intermediates and delineated the biosynthetic routes contributing to *N*. *fowleri* sterol homeostasis. An array of sterol molecules suggests that two different sterol types, ergosterol-like and cholesterol-like sterols, co-exist and may be dynamically regulated in *N*. *fowleri*. Disruption of sterol biosynthesis by drug candidates was detrimental to *N*. *fowleri*. Drugs targeting three different enzymatic steps resulted in growth inhibition effect. This effect was amplified when drugs targeting different enzymes were combined. The observed synergistic effect of these drugs lowers the individual drug concentrations required for biological activity including delivering anti-PAM drugs across the blood-brain-barrier.

## Introduction

*Naegleria fowleri* and its non-pathogenic relatives, *Naegleria gruberi* and *Naegleria lovaniensies*, belong to the genus *Naegleria*, class Heterolobosea, which is characterized by a capacity for quick differentiation from an amoeboid to a flagellate form. These amoebae feed mostly on bacteria, but can also act as opportunistic pathogens causing infections of the central nervous system (CNS) of humans and other animals. *N*. *fowleri* is the only species of the genus known to cause a severe primary amebic meningoencephalitis (PAM) in humans.[1] *Naegleria* occur in three forms–a cyst, a trophozoite (amoeboid), and a biflagellate. The trophozoite is the only feeding and reproductive stage of *Naegleria* spp., as well as the only one found in infected brain tissue[2], while the flagellate form was detected in the cerebrospinal fluid (CSF)[3]. PAM due to *N*. *fowleri* has a worldwide distribution although it occurs most frequently in tropical areas and during hot summer months.[4]

*N*. *fowleri* infection is problematic due to the rapid onset and destructive nature of the disease as well as to the lack of established success in treatment.[5] Until recently, no more than a dozen patients out of ~350 reported PAM cases worldwide have been treated successfully with Amphotericin B (AmpB), either alone or in combination with other drugs.[6–9]. The investigational anti-cancer and anti-leishmaniasis agent miltefosine[10] showed promise, but not all patients who received miltefosine as part of their treatment regimens survived. In 2013, two patients survived out of three treated with miltefosine, but one of the survivors had permanent brain damage.[11] In 2016–2017, two more patients receiving miltefosine survived out of 9 diagnosed with PAM. The lack of a single, proven, evidence-based treatment of PAM with a high probability of cure stimulates a need to further study *Naegleria* biology in order to understand molecular mechanisms maintaining *N*. *fowleri* homeostasis throughout different developmental stages and dietary conditions.

Sterols are an important class of lipids essential in all eukaryotes. It is assumed that the last eukaryotic common ancestor (LECA) already synthesized sterols.[12] Eukaryotes that lost the ability to synthesize sterols, *e*.*g*., insects, worms and most marine invertebrates, have to obtain them from food. Sterols are involved in both intra- and intercellular signaling and in the organization of membranes. They affect fluidity and permeability of membranes and are major players in the formation of lipid rafts, regions of reduced fluidity formed by the close association of sterols with sphingolipids. [13–15] In unicellular organisms, lipid rafts control endocytosis, vesicle trafficking, motility, and cell signaling and have been shown to regulate adhesion to host cells and control virulence (reviewed in [16]). Ergosterol, a fungal and kinetoplastid sterol, was shown to promote formation of the lipid rafts.[17]

By DNA sequence, *Naegleria* are close to kinetoplastids, however, in contrast to the lanosterol precursor in kinetoplastids,[18] *de novo* biosynthesis of ergosterol in amoebae occurs from cycloartenol, a precursor typical of photosynthetic organisms, ie., algae and plants.[19–21] Disruption of sterol biosynthesis by small-molecule inhibitors targeting CYP51 is detrimental for *N*. *fowleri* trophozoites, suggesting that ergosterol biosynthesis is essential for amoeboid survival.[22] Among the enzymes constituting the sterol biosynthetic pathway in eukaryotes, several targets have been studied for the development of therapeutic or agricultural agents. For instance, the HMG-CoA reductase inhibitors, known as statins, are drugs used for lowering serum cholesterol. Farnesyl diphosphate synthase (targeted by bisphosphonates), squalene synthase (aryloxyethyl thiocyanate and quinuclidine derivatives), squalene epoxidase (terbinafine), oxidosqualene cyclase (pyridinium-ion mimetics), sterol 14α-demethylase (CYP51) (azoles), sterol C24-methyltransferase (SMT) (arylguanidines, azasterols), and sterol Δ^8^−Δ^7^ isomerase (ERG2) (morpholines) have been validated as drug targets to treat fungal infections in humans and plants.

In this work, we have assessed the sterol biosynthesis pathway in *N*. *fowleri* downstream of CYP51 by GC-MS analysis of the metabolic intermediates accumulated in trophozoites in response to the inhibitors with known mechanisms of action (MOA). Using inhibitors as the molecular probes, we chemically validated SMT and ERG2 as essential enzymes in *N*. *fowleri*. *In vitro* growth inhibition effect were observed for inhibitors of each mechanistic group applied individually. When applied in combination, inhibitors with different MOA produced synergistic effects. The biosynthetic cascade reconstituted in *N*. *fowleri*, based on the flux of the metabolic intermediates, indicates that the steroidogenic pathway bifurcates into ergosterol and cholesterol arms. The observed sterol profile suggests a possibility of dynamic modulation of sterol homeostasis in *N*. *fowleri* in response to environmental factors or dietary conditions.

## Results and discussion

### Sterol biosynthesis enzymes in *Naegleria*

Biosynthetically, sterols are derived from isopentenyl diphosphate (IPP), the precursor of squalene and its downstream cyclized products. The first step leading to sterols adds an oxygen to squalene resulting in squalene epoxide, which is then cyclized in a second step either to lanosterol or to cycloartenol. Enzymes constituting the core sterol biosynthesis pathway in eukaryotes largely exhibit strong conservation in amino acid sequences; however, the pathway architecture and substrate specificity vary.[23] Steroidogenesis in the non-pathogenic environmental *Naegleria* species, *N*. *gruberi* and *N*. *lovaniences*, was assessed in the pre-genomic era.[20] Following up on results from earlier studies[22], we have now interrogated the sterol biosynthesis pathway in *N*. *fowleri*. *Naegleria* steroidogenic enzymes were identified by sequence homology to the yeast, human and plant orthologues. They are characterized by high intra-genus sequence similarities and lower similarity to the human counterparts, the latter ranging from 32 to 56% for homologous enzymes (**S1 Table)**. The Δ^24^-reduction and Δ^8^−Δ^7^ -isomerization steps are performed by non-homologous enzyme pairs (<20% sequence identity), ERG4 and ERG2 in fungi, DHCR24/DWF1 and EBP/HYD1 in vertebrates/land plants respectively. The *Naegleria* enzymes are homologous to fungal orthologues, suggesting that both can be exploited therapeutically.

The 3-keto-steroid reductase and C22-desaturase could not be identified in *Naegleria* by homology with yeast, human or plant counterparts. The *Naegleria* 3-keto-steroid reductase that catalyzes the last of the three steps required to remove two C-4 methyl groups may be homologous to yet unknown C-3 ketoreductase in land plants.[12] A protein with similarity to CYP710A, equivalent to 22-desaturase in higher plants, is present in *N*. *gruberi* (XP_002678320.1), but its primary sequence is closer to that of CYP51. In animals, lack of 22-desaturase is a typical feature. The 24-alkylation performed by ERG6 is present in *Naegleria* but absent in animals.

### The sterol metabolic network in *N*. *fowleri*

**Fig 1:**
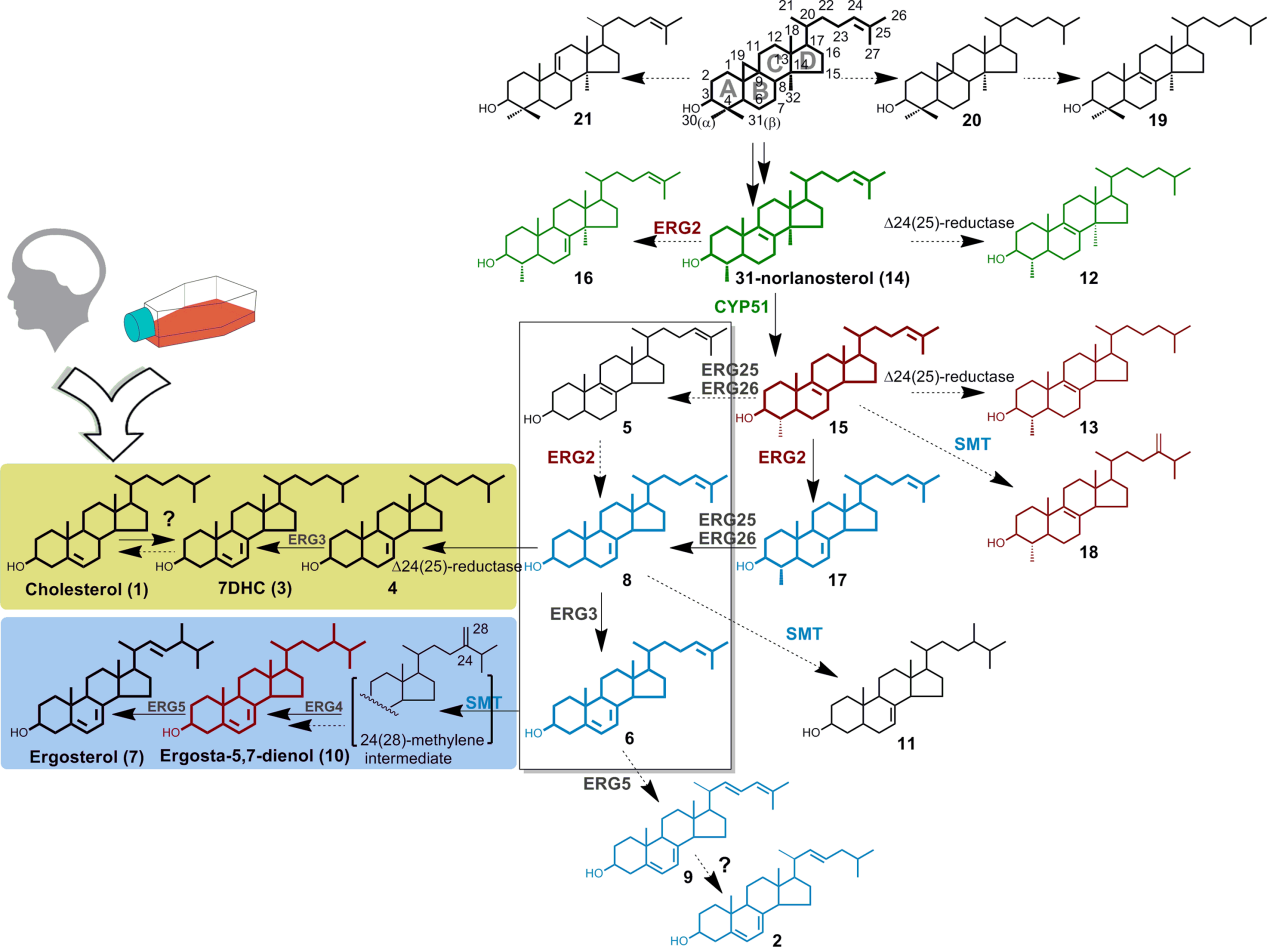
Sterol metabolic network in *N*. *fowleri*. The pathway is reconstituted based on the metabolites observed in the untreated *N*. *fowleri* (shown in bold) and treated with the CYP51 (shown in green), SMT (in blue) and ERG2 (in dark red) inhibitors. Metabolites not observed in the wild-type *N*. *fowleri* are depicted in thin lines. The cholesterol arm of the pathway is highlighted by the yellow-green background, the ergosterol arm is highlighted by the blue background. Enclosed in frame are potential SMT substrates. Enzymes interrogated in these studies are labeled in colors corresponding to the accumulating metabolites. Numbering of atoms in sterol molecule is adapted from Nes and Venkatramesh, 1994.[41] Sterol labelling is as in **Table 1.** ERG enzyme nomenclature is according to the convention for *S*. *cerevisiae*.

We used sterol biosynthesis inhibitors with different MOA to (**i**) interrogate the flux of sterol intermediates, (**ii**) access the physiological requirement for specific sterols and (**iii**) identify enzymatic chokepoints essential to trophozoite growth. The sterol biosynthetic cascade, reconstituted in *N*. *fowleri* based on the flux of the metabolic intermediates, is consistent with the earlier data [20, 22] that sterol biosynthesis in *Naegleria* proceeds from cycloartenol (**22**) via 31-norlanosterol (**14**) to ergosterol (**7**) (**Fig 1**). Cholesterol (**1**), 7-dehydrocholesterol (7DHC) (**3**), ergosterol (**7**) and its biosynthetic precursor ergosta-5,7-dienol (**10**) are the most abundant sterols dominating lipid extracts of the non-inhibited *N*. *fowleri* and *N*. *gruberi* trophozoites, whereas 4-monomethylsterols, 4,4-dimethyl intermediates and 14α-methylsterols are sparsely represented (**Table 1**).

**Table 1:** Sterol flux in *N*. *fowleri*.

			Non-inhibited		CYP51	SMT			Sterol Δ^8^−Δ^7^−isomerase	
##	Metabolites^a^	RRT^b^	*NG*	*NF*^*c*^	Posaconazole^*c*^	Epiminolanosterol	Abafungin	25-Aza	Tamoxifen	AY9944
1	Cholesterol	1	7.6	25.6	26.0	15.6	2.0	12.7	3.0	9.5
	***4-Desmethylsterols (w/o cholesterol)***		**77.6**	**62.9**	**23.8**	**61.9**	**88.8**	**74.5**	**71.9**	**54.6**
2	Cholesta-5,7,22-trienol	1				3.3	4.3	2.9		
3	7-Dehydrocholesterol (7DHC)	1.04	10.8	12.5	3.9	9.9	13.8	12.9	4.1	7.5
4	Lathosterol	1.06		0.8			0.2			
5	Zymosterol	1.07		0.4		0.1		0.1		
6	Cholesta-5,7,24-trienol	1.11				30.8	30.0	38.8		
7	Ergosterol	1.11	35.5	26.7	14.2	7.7	20.7	8.8	24.3	22.8
8	Cholesta-7,24-dienol	1.12				1.6	5.5	2.8		
9	Cholesta-5,7,22,24-Tetraenol	1.20				6.9	8.6	5.3		
10	Ergosta-5,7-dienol	1.21	31.1	21.3	5.7	1.6	5.7	2.9	43.5	24.3
11	Ergost-7-enol	1.22	0.2	1.2						
	***4-Monomethylsterols***		**2.1**	**6.8**	**48.5**	**20.8**	**8.0**	**10.5**	**13.9**	**29.7**
12	4α,14α-Dimethylcholest-8-enol	1.08	0.1	1.1	15.5					
13	4α-Methylcholest-8-enol	1.11		3.5	0.4	7.6	7.2	2.6	10.4	5.7
14	31-Norlanosterol	1.14	2.0	2.2	29.7	0.5		1.4		
15	4α-Methylcholesta-8,24-dienol	1.18				9.1	0.4	1.5	3.5	19.1
16	Δ^7^-31-norlanosterol	1.24			2.9					
17	4α-Methylcholesta-7,24-dienol	1.26				3.6	0.4	5.0		2.9
18	4α-Methylergosta-8,24(28)dienol	1.31								2.0
	***4*,*4-Dimethylsterols***		**9.6**	**3.3**	**1.0**	**0.9**	**0.6**	**0.9**	**2.3**	**0.8**
19	Lanostanol	1.30	0.5	0.2	0.2	0.4	0.5			
20	Cycloartanol	1.32	6.9	1.1						
21	Parkeol	1.40	0.3		0.1	0.1		0.3		
22	Cycloartenol	1.41	1.9	2.0	0.7	0.4	0.1	0.6	2.3	0.8
	**Other sterols**		**3.1**	**1.4**	**0.7**	**0.8**	**0.6**	**1.4**	**8.9**	**5.4**

^a^All listed sterol structures are shown in **Fig 1**. The metabolites were quantified based on the total ion current peak areas of each sterol. NF–*N*. *fowleri*, NG–*N*. *gruberi*

^b^Relative retention time compared to cholesterol

^c^Previously published data[22] used here as a comparative companion in the context of the whole pathway.

Given structural similarity of sterol substrates, enzyme specificity is controlled kinetically rather than thermodynamically. Disruption of specific steps gives rise to the uncommon intermediates that are enzymatically converted to the end-products not operational in the wild-type organism. As reported previously,[22] upon treatment of *N*. *fowleri* with posaconazole, the cumulative content of the 14α-methylsterols, including **12**, **14**, **16**, **19**, **20**, **21** and **22**, increased from 6.5% to 49.1%, with the major intermediate being 31-norlanosterol (**14**) followed by its C24-C25 hydrogenated product, 4,14-dimethylcholest-8-enol (**12**). Posaconazole data reported elsewhere[22] are included in **Table 1**as a comparative companion in the context of the whole pathway. Given the unchanged content of 4,4-dimethylsterols in the CYP51 inhibitor-exposed samples, removal of the 4β-methyl group is unaffected by the blocking of CYP51 activity and likely occurs prior to the C-14 demethylation; removal of the 4α-methyl group occurs downstream to CYP51. The C-4 demethylation in two nonconsecutive steps is a feature of land plants.[12] In this work, by using molecular probes with the corresponding MOA, we mapped enzymatic steps downstream of CYP51, including sterol C8→C7-double bond isomerization, catalyzed by ERG2, and sterol C24-methylation, catalyzed by SMT (ERG6).

In an ERG2 inhibitor AY9944-treated *N*. *fowleri*, the 4α-methylcholesta-8,24-dienol (**15**) content increased from zero to 19.1% suggesting that the 4α-demethylation follows the Δ^8^−Δ^7^ -isomerization and preferably occurs on 4α-methylcholesta-7,24-dienol (**17**). The treatment of *N*. *fowleri* with another ERG2 inhibitor, tamoxifen, a non-steroidal estrogen receptor modulator, also resulted in an increase of the ergosta-5,7-dienol (**10**) content from 21.3% to 43.5% (**Fig 1**, **Table 1**), suggesting an inhibition of 22-desaturation performed by a yet unknown *Naegleria* enzyme.

**Fig 2:**
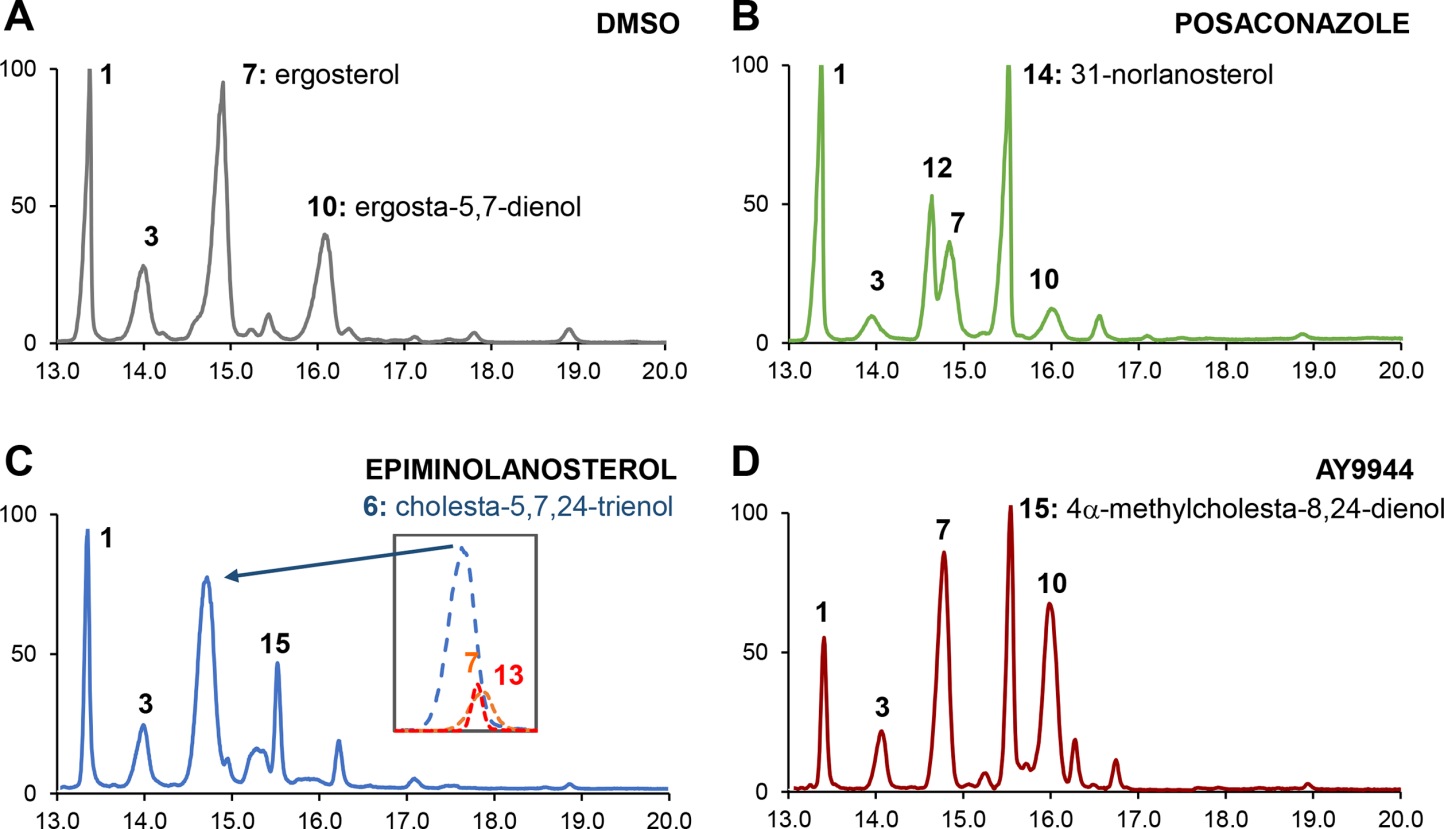
Lipid analysis by GC-MS. Gas chromatography of the total sterol fractions derived from *N*. *fowleri* trophozoites treated with (**A**) 0.5% DMSO, (**B**) 0.2 μM posaconazole, (**C**) 5.4 μM epiminolanosterol, and (**D**) 5.6 μM AY9944. Peaks are labeled with numbers corresponding to metabolites listed in **Table 1**. Insert in (**C**) shows deconvolution of the major peak resulting from an overlap of three different sterols: **6**, m/z = 349; **7**, m/z = 363 and **13**, m/z = 400. The color scheme is the same as in **Fig 1**: CYP51 inhibitor (shown in green), SMT inhibitor (in blue) and ERG2 inhibitor (in dark red).

Exposure of *N*. *fowleri* to the SMT inhibitors 24,25-epiminolanosterol (epiminolanosterol), abafungin and 25-azacycloartenol (25-Aza), led to high accumulation of cholesta-5,7,24-trienol (**6**) (≥30% of total sterols), an intermediate undetectable in the wild-type or posaconazole-inhibited *N*. *fowleri* (**Table 1**, **Fig 2**). This finding made three intermediates, **5**, **8** and **6**, potential candidates for endogenous SMT substrates in *N*. *fowleri*. All three contain a Δ^24(25)^ olefin group that allows C-methylation with formation of the transient 24-methylene intermediate that ultimately is converted to the ergosterol-like physiological end-products (**Fig 1**).[24, 25] In the SMT-inhibited *N*. *fowleri*, the end-products **9** and **2**, not operational in the wild-type *N*. *fowleri*, were also formed. Accumulation of cholesta-5,7,22,24-tetraenol (**9**) and cholesta-5,7,24-trienol (**2**), the end products representing a dysfunctional carbon flux, was previously reported in yeast carrying an ERG6 mutant defective in SMT activity.[26]

### Functional analysis of *Naegleria* sterol C24-methyltransferase (SMT)

To identify what configuration of the double-bond(s) in the sterol B-ring favors *Naegleria* SMT catalysis, the studies using recombinant enzyme and individually isolated sterol intermediates were conducted. In the *N*. *gruberi* genome, two candidate genes encoding SMT (77.6% sequence identity) were identified (**S1 Table**), which resemble those of higher plants in primary sequence (~50% identity). Higher plants have two SMT enzymes that use different substrates to give either C24-methylated or C24-ethylated phytosterols. *N*. *fowleri* has a single gene encoding SMT; no 24-ethylated sterols were detected in the lipid extracts of any *Naegleria* species. *N*. *gruberi* SMT (XP_002680047.1) sharing highest sequence identity (86%) with the *N*. *fowleri* counterpart, has been expressed in *Escherichia coli* and recombinant protein was characterized for substrate specificity. From the *in vitro* reconstitution of the SMT catalytic activity, among thirteen sterols tested, **5** (zymosterol) and **6** (cholesta-5,7,24-trienol) were equipotent as SMT substrates (set at 100%), while **8** (cholesta-7,24-dienol) showed 67.2% convertion (**Table 2**).

**Table 2:** Conversion of different sterols by *N*. *gruberi* SMT.

Substrates	Relative Activities (%)
fecosterol	0.0
24-methylenelophenol	0.0
zymosterol (**5**)	100.0
cholesta-5,7,24-trienol (**6**)	100.0
cholesta-7,24-dienol (**8**)	67.2
14α-methylzymosterol	29.0
desmosterol	17.10
4α-methylzymosterol	9.4
31-norlanosterol (**14**)	2.7
cycloartenol (**22**)	0.0
24-methylenecycloartenol	0.0
lanosterol (**19**)	0.0
obtusifoliol	0.0

### An origin of cholesterol in *Naegleria*

Remarkably, in addition to the ergosterol ‘arm’ (highlighted blue in **Fig 1**), a cholesterol arm, characterized by reactions **8→4→3→1** (highlighted orange in **Fig 1**), emerged in the metabolic flux studies. Together with the high cholesterol content, this observation raised a question about the origin of cholesterol: *de novo* biosynthesis or scavenging from the growth medium. To answer this question, we have conducted the ^13^C-labeling studies in *N*. *gruberi* grown in the ATCC1034 medium supplemented with U-^13^C glucose to the final concentration of 5% (w/v). Incorporation of the ^13^C-carbon was monitored by GC/MS; a percent of the ^13^C enrichment for each sterol was calculated from the isotopic distribution of the molecular mass using IsoCor software.[27] As expected, cycloartenol, ergosta-5,7-dienol and ergosterol made by amoeba biosynthetically, had the highest ^13^C-isotope content (**Table 3**). At the same time, cholesterol was unlabeled, proving its exogenous origin in cultured *N*. *gruberi* trophozoites. Remarkably, ^13^C-isotope content in 7-dehydrocholesterol (**3**) fall between the ergosterol and cholesterol values, suggesting dual origin of this intermediate in both the *de novo* biosynthesis and via a reverse reaction catalyzed in other species by cholesterol C-7 desaturase. Cholesterol 7-desaturase is a critical enzyme in insects and nematodes involved in the synthesis of steroid hormones (DAF-36).[28] These lineages lack sterol biosynthesis capacity and rely solely on cholesterol uptake from the diet. Both *N*. *fowleri* and *N*. *gruberi* genomes encode a putative DAF-36-like protein (**S1 Table**).

**Table 3:** Isotopic enrichment of *N*. *gruberi* sterols incubated with 50 mM U-^13^C-glucose.

Sterols	Molecular Formula	^13^C-content in normal medium (%)	^13^C-content in U^13^C-glucose medium (%)
Cholesterol	C_27_H_46_O	1.07	1.06
Cholesta-5,7-dienol	C_27_H_44_O	1.11	2.46
Ergosterol	C_28_H_44_O	1.06	4.02
Erogsta-5,7-dienol	C_28_H_46_O	1.11	4.03
Cycloartenol	C_30_H_50_O	1.15	4.15

The dual origin of 7DHC extends the cholesterol arm all the way from **8→4→3** (**Fig 1**). The last **3→1** step separating *N*. *fowleri* from making cholesterol *de novo*, the reduction of the C7-C8-double bond in 7-dehydrocholesterol usually performed by Δ^7^-dehydrocholesterol reductase, remains unobserved. Candidates for this enzymatic role are present in both *N*. *fowleri* and *N*. *gruberi* genomes (**S1 Table**). A possibility of the pathway bifurcation downstream of **8** suggests a “switch” between the C24-methylation and Δ^24^-reduction steps (**Fig 1**) committing *Naegleria* to either ergosterol or cholesterol biosynthesis, respectively. We hypothesize that the switch may be used to maintain dynamic sterol homeostasis over the course of the *N*. *fowleri* life-cycle.

### Anti-proliferative and synergistic effects of sterol biosynthesis inhibitors

**Fig 3:**
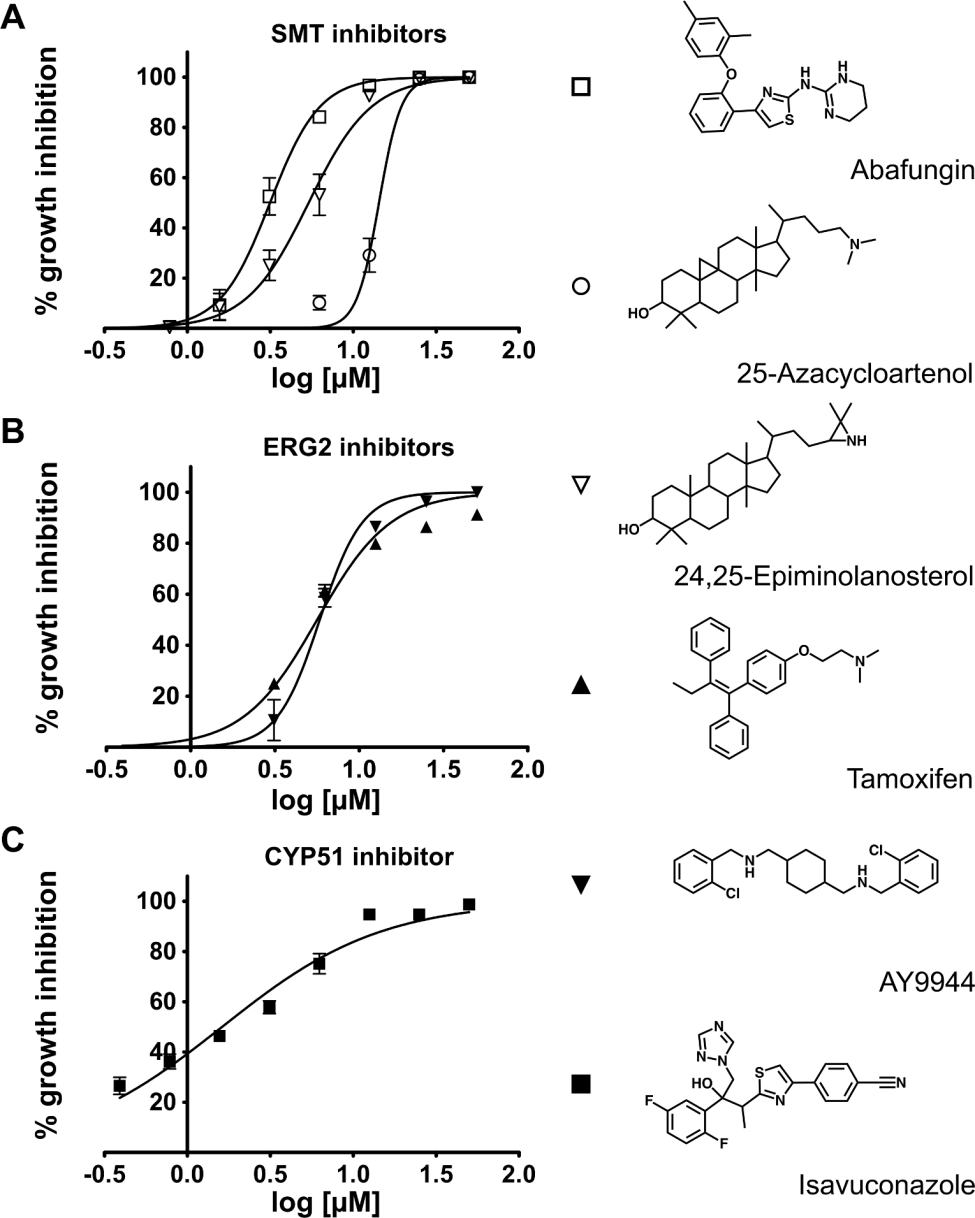
Dose-response growth inhibition of *N*. *fowleri* trophosoites. Dose-response curves are shown for (**A**) SMT inhibitors: abafungin, 25-azacycloartenol and 24,25-epiminolanosterol, (**B**) ERG2 inhibitors: tamoxifen and AY9944, (**C**) CYP51 inhibitor isavuconazole.

The growth inhibition of free-living amoebae by the sterol biosynthesis inhibitors has been reported previously. Thus, the effect of the agricultural systemic fungicides, tridemorph and fenpropimorph, which target both sterol Δ^8^−Δ^7^ -isomerase and Δ^14^-reductase,[29] has been reported in *Acanthamoeba polyphaga*, *N*. *lovaniensis* and *N*. *gruberi* in the mid 80’s.[20, 21] More recently, growth inhibition of *Acanthamoeba castellanii* and *A*. *polyphaga* was demonstrated by the pharmaceutical sterol biosynthesis inhibitors, itraconazole and voriconazole, targeting CYP51.[30] Finally, posaconazole and itraconazole were found superior to AmpB, a cornerstone of PAM therapy and a standard of care for CNS infections caused by molds, against *N*. *fowleri* trophozoites *in vitro*.[22] In this work, we demonstrated that blocking enzymatic steps downstream of CYP51, leads to a decline of ergosterol and/or accumulation of the end-products and metabolic intermediates incompatible with *N*. *fowleri* survival *in vitro*. The anti-proliferative effects of compounds were assessed using methods previously described.[7, 22] The EC_50_ values of the individual inhibitors deduced from the dose-response curves (**Fig 3**) are summarized in **Table 4**. For drug compatibility in combination experiments, all compound stocks were prepared in DMSO, which negatively affected the isavuconazole and posaconazole EC_50_ values compared to previously determined for the SBE-β-CD-solubilized drug.[22] The *in vitro* potency of all tested sterol biosynthesis inhibitors exceeded that of miltefosine (EC_50_ of 54.5 μM), an investigational drug currently recommended by the U.S. Centers for Disease Control and Prevention for the treatment of PAM.

**Table 4:** Targeting *N*. *fowleri* with inhibitors of known MOA.

Inhibitors	EC_50_, ^μ^M *N*. *fowleri*
**CYP51 (ERG11)**	
Posaconazole^a^	≤0.01[22]
Posaconazole	0.2±0.08
Isavuconazole^a^	0.1[22]
Isavuconazole	1.6±0.03
**SMT (ERG6)**	
Abafungin	3.1±0.02
Epiminolanosterol	5.4±0.03
25-Azacycloartenol	14.3±0.04
**Sterol Δ^8^−Δ^7^ isomerase (ERG2)**	
Tamoxifen	5.8±0.04
AY9944	5.6±0.03
**Human σ_1_ receptor**	
Fluoxetine (Prozac)	31.8±0.01
Fluvoxamine (Luvox)	42% at 50 μM
Citalopram (Celexa)	21% at 50 μM
Dextromethorphan (DXM)	20% at 50μM
**Standards of care**	
AmpB^a^	0.10±0.01[22]
Miltefosine	54.4±0.01[22]

^a^solubilised in SBE-β-CD

**Fig 4:**
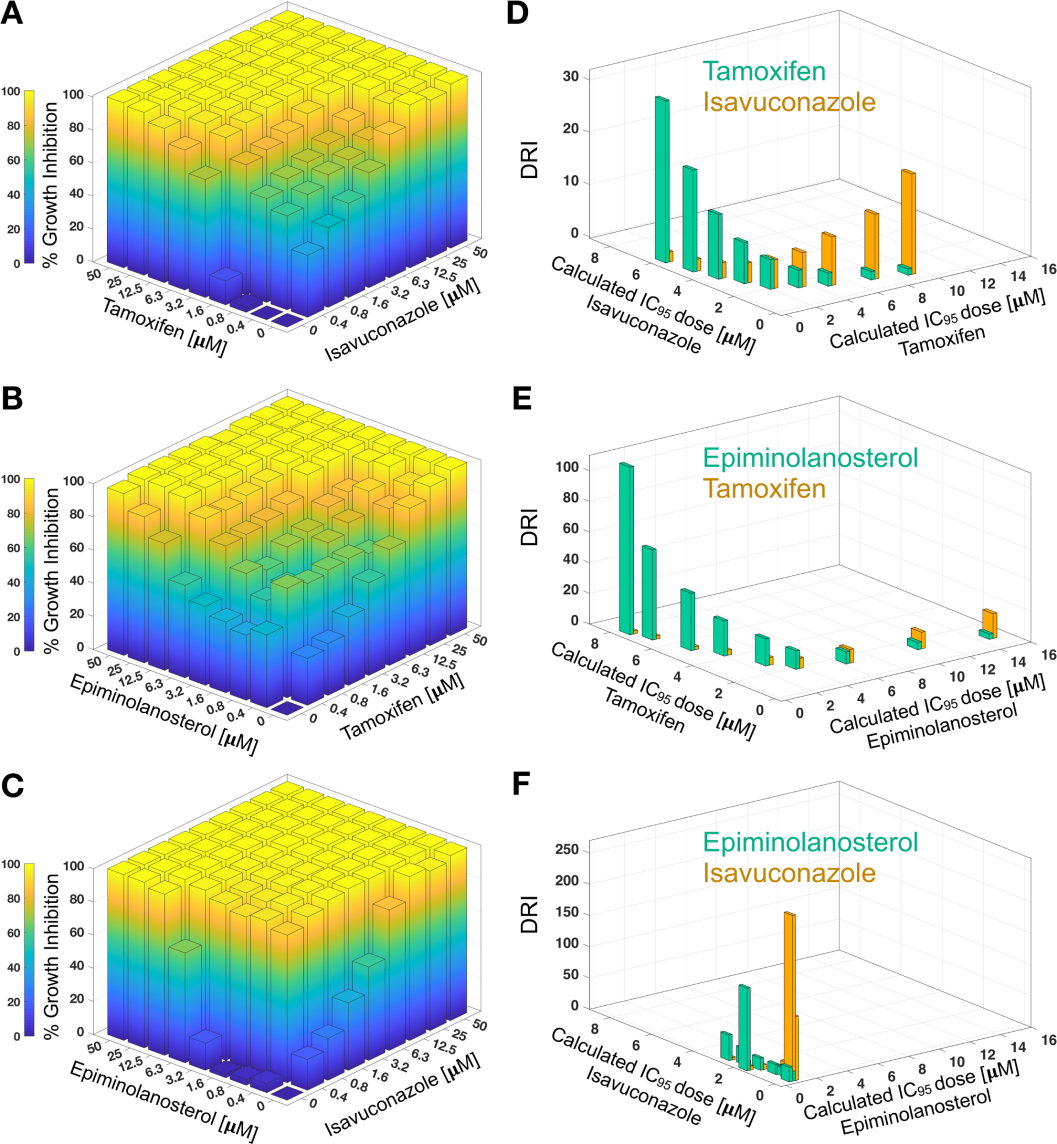
Growth inhibition of *N*. *fowleri* as a function of drug combinations. Heat maps show growth inhibition of *N*. *fowleri* by drug combinations: tamoxifen and isavuconazole (**A**), epiminlanosterol and tamoxifen (**B**), and epiminolanosterol and isavuconazole (**C**). Corresponding isobolograms (**D, E, F**) show the mean values of dose reduction index (DRI) ploted against calculated inhibitor doses required to achieve 95% growth inhibition. Standard deviations for each parameter mean value are shown in **S2 Table**.

Blocking two essential enzymes in the ergosterol pathway is more detrimental for unicellular organisms than inhibiting one.[31] This synergistic effect is due to more efficient depletion of an essential end product(s) and/or redirection of substrate flow to produce intermediates and end-products harmful for key steroidogenic enzymes or membrane structures. In this work, we have demonstrated synergy between the CYP51 (isavuconazole), SMT (epiminolanosterol) and sterol ERG2 (tamoxifen) inhibitors paired in three different combinations as specified in **Fig 4**. Using classical isobolograms, combination indices (CI) and dose reduction indices (DRI) were calculated from the experimental data (**Fig 4A, 4B and 4C**) as described by Chou and Talalay (**S2 Table**). [32, 33] The isobolograms indicate synergy for all three drug pairs in achieving 95% of *N*. *fowleri* growth inhibition with 2- to 19-fold dose reduction for isavucanazole and 1- to 19-fold dose reduction for tamoxifen (**Fig 4D**); 3- to 109-fold dose reduction for epiminolanosterol and 2- to 16-fold dose reduction for tamoxifen (**Fig 4E**); and finally, the highest synergy with 4- to 265-fold dose reduction for isavucanazole and 13- to 132-fold dose reduction for epiminolanosterol is indicated for the isavuconazole-epiminolanosterol pair (**Fig 4F**).

**Fig 5:**
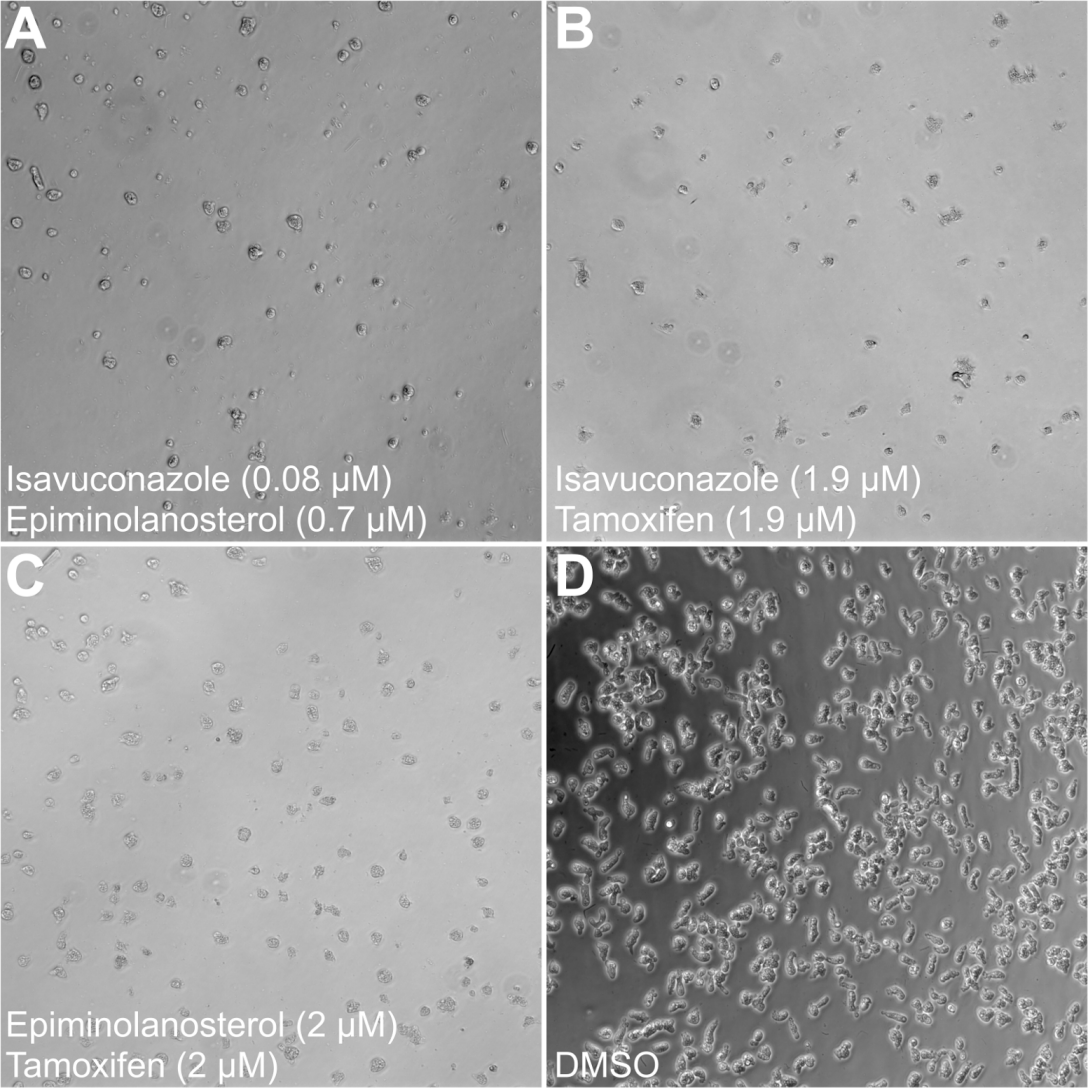
Synergistic effect of drugs at low concentrations. The phase contrast microscope images show *N*. *fowleri* trophozoites treated for 48 hours with (**A**) 0.08 μM of isavuconazole and 0.7 μM of epiminolanosterol, (**B**) 1.9 μM equimolar concentrations of isavuconazole and tamoxifen, (**C**) 2 μM equimolar concentrations of epiminolanosterol and tamoxifen, and (**D**) 0.5% DMSO, which served as a negative control. The inhibitor-treated *N*. *fowleri* cells visible in the microscope field are rounded, much smaller in size and not viable, whereas DMSO-treated cells are irregularly shaped with visible cytoplasm. Magnification, ×20.

The drug combinations with the highest predicted synergy were analyzed microscopically for their effect on *N*. *fowleri* growth and morphology (**Fig 5**). The isavuconazole-epiminolanosterol pair, combined at concentrations of 0.08 μM and 0.7 μM, respectively, (CI = 0.07) (**Fig 5A**), the isavuconazole-tamoxifen pair, combined at 1.9 μM equimolar concentrations (CI = 0.36) (**Fig 5B**), and epiminolanosterol and tamoxifen pair, combined at 2 μM equimolar concentrations (CI = 0.38) (**Fig 5C**), completely inhibited the growth and led to the changed morphology and death of the majority of *N*. *fowleri* cells in 48 h of drug exposure, whereas DMSO-treated control cells grew and appeared normal (**Fig 5D**).

### New therapeutic targets and blood-brain permeability of drugs

**Fig 6:**
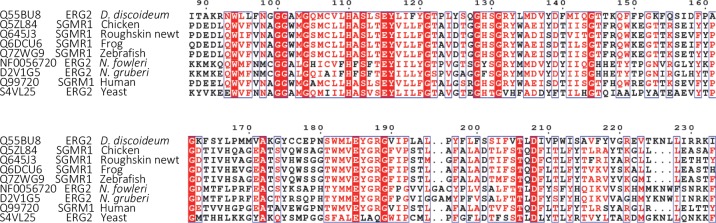
Sequence alignments between the catalytic domain of sterol Δ^8^−Δ^7^ -isomerase (ERG2) and a ligand binding site of non-opioid σ_1_ receptor (SGMR1) demonstrate high sequence similarity. Accession numbers in UniProt or AmoebaDB are provided for each sequence. Numbering is according to the *Dictyostelium discoideum* ERG2 sequence.

Drug ‘repurposing’ is a relevant and cost effective strategy for utilizing approved drugs for the treatment of rare diseases. The requirement of brain penetrance narrows down the pool of the FDA-approved candidates for the treatment of PAM. Only a few diseases of the brain like depression, affective disorders, chronic pain, and epilepsy respond to small-molecule therapy. The reason for the paucity of small-molecule drugs is that 98% of them do not cross the blood-brain barrier (BBB).[34, 35] Validation of sterol Δ^8^−Δ^7^ -isomerase (ERG2) as a new potential molecular drug target in *N*. *fowleri* provides a link to structurally diverse brain-penetrant small-molecule drugs targeting human non-opioid σ_1_ receptor sharing 30% sequence identity and 60% sequence homology to the catalytic domain of the *N*. *fowleri* sterol Δ^8^−Δ^7^ -isomerase (**Fig 6**). The non-opioid σ_1_ receptor is implicated in human CNS conditions such as addiction, amnesia, pain and depression.[36]

We have tested four non-selective serotonin reuptake inhibitors (SSRIs) and σ1 receptor agonists with reported affinity to human σ1 receptors, fluvoxamine (Luvox), fluoxetine (Prozac), citalopram (Celexa) and dextromethorphan (DXM), [37, 38] for growth inhibition against *N*. *fowleri*. All four drugs demonstrated effect at the highest tested concentration (50 μM). Fluoxetine, the well-known antidepressant, was the most potent in this group (**Table 4**). Potency of fluoxetine (EC_50_ of 32 μM) exceeded that of miltefosine (54 μM). Other structurally diverse brain-penetrant small-molecule drugs targeting this human receptor will now be screened for anti-*Naegleria* activity to identify candidates for animal model studies.

### Summary

The sterol biosynthetic pathway in *N*. *fowleri* displays a mixture of canonic features peculiar to different domains of life: lower eukaryotes, land plants, invertebrates and vertebrates. In addition to the cycloartenol→ergosterol biosynthetic route, our data suggest a route leading to *de novo* cholesterol biosynthesis with a single, yet to be experimentally observed, enzymatic step separating *N*. *fowleri* from making cholesterol *de novo*. *N*. *fowleri* also utilizes cholesterol obtained from diet by converting it into 7-dehydrocholesterol, a feature typical to the eukaryote lineages (*e*.*g*., insects, worms and marine invertebrates) that lost the ability to produce sterols from squalene. Hypothetically, a “switch” between ergosterol and cholesterol sterol types may occur in *Naegleria*, *e*.*g*., during encystation/excystation, to sustain environmental challenges by forming a viable cyst. By demonstrating the amoebicidal effect of the sterol biosynthesis inhibitors with different MOA, we validated multiple, potentially druggable, molecular targets in *N*. *fowleri*, linking the anti-*Naegleria* drug discovery to the existing small-molecule drugs targeting other human diseases. Synergy between alternative drug-targets is of interest for the treatment of PAM, as lower drug concentrations are required to achieve the biologic effect. Reducing therapeutic concentrations may, in part, alleviate the limitation imposed by the need of delivering anti-PAM drugs accross the blood-brain-barrier.

## Materials and methods

### Ethics statement

Research performed at UC San Diego was conducted in compliance with the Animal Welfare Act and other federal statutes and regulations relating to animals and experiments involving animals and adheres to the principles stated in the Guide for the Care and Use of Laboratory Animals, National Research Council, 2011. The facility where this research was conducted is fully accredited by the Association for Assessment and Accreditation of Laboratory Animal Care International. Animal research was conducted under approved protocol S14187 from the Institutional Animal Care and Use Committee, University of California, San Diego. Euthanasia was accomplished by CO_2_ inhalation or by sodium pentobarbital overdose (60 mg/kg), followed by cervical dislocation. These methods of euthanasia have been selected because they cause minimal pain and distress to animals, are relatively quick, and do not adversely impact interpretation of the results of studies. All methods are in accord with the recommendations of the Panel on Euthanasia of the American Veterinary Medical Association.

### Compounds

The inhibitors tested were purchased from commercial vendors except epiminolasosterol and 25-azacycloartenol which were synthesized in Dr. Nes’ laboratory; the structure of the sterol was authenticated by both the GC-MS and NMR methods. AY9944 and abafungin were from Santa Cruz Biotechnology (SC-202965 and SC-474844); tamoxifen was from Sigma (T5648), isavuconazole was from StruChem (SC-98350), fluvoxamine (J90043), fluoxetine (C845), citalopram (K750) and dextromethorphan (X3585) were from AK Scientific.

### *N*. *fowleri* strain maintenance

The *N*. *fowleri* KUL strain used in these studies was acquired from ATCC in 2015; aliquoted stock is maintained deep frozen in liquid nitrogen. *N*. *fowleri* amoebae maintained axenically (in culture) are weakly virulent. To maintain highly virulent status, *N*. *fowleri* strain is passaged every six months in mouse brain. For that, four week-old male Balb/c mice are inoculated by intranasal instillation of 10 μl of 3x10^4^ *N*. *fowleri* trophozoites axenically cultured at 37°C in Nelson’s medium supplemented with 10% fetal bovine serum, resulting in 100% animal mortality at day 7. Female mice are less susceptible to *N*. *fowleri* infection, therefore only male mice are used in this model. At the time point when clinical signs of infection are apparent, the mice are sacrificed and the brains are harvested to propagate *N*. *fowleri* axenically for the next few months. The animals and tissues infected with *N*. *fowleri* are handled in the certified Biosafety Level-2 (BSL-2) environment according to the UCSD Institutional Animal Care and Use Committee guidelines and approved animal protocol. Safety glasses, gloves, mask, disposal gowns, shoe covers and head cover are worn. *N*. *fowleri* strain is handled in a biosafety cabinet mounted in the BSL-2 environment.

### *N*. *fowleri* growth inhibition assay

To determine EC_50_ values, test compounds were tested for dose-response against *N*. *fowleri* trophozoites axenically cultured in Nelson’s medium supplemented with 10% fetal bovine serum at 37°C;[39] all the experiments were performed in triplicate using trophozoites harvested during the logarithmic phase of growth.[40] Drug concentration ranges of 0.4–50 μM and 0.008–25 μM were generated by transferring 0.5 μl of serially diluted compounds to a corresponding well of the 96-well plate followed by addition of 99.5 μl of *N*. *fowleri* trophozoites (10,000 amoebae). Assay plates were incubated for 48 h and cell viability was determined by the CellTiter-Glo Luminescent Cell Viability Assay.[7, 40] The experiments using trophozoites were conducted in a biosafety cabinet following the BSL-2 procedures as specified in the UCSD Biosafety Practices Guidelines.

### GC-MS analysis of the *Naegleria* sterols

Twenty or fifty million *N*. *gruberi* or *N*. *fowleri* trophozoites per sample were exposed for 24 h to 0.5% DMSO or an inhibitor at EC_50_ concentration prior to lipid extraction. Sterols were analyzed by the use of GC-MS, wherein the lipids extracted from cells grown in the presence of non-lethal concentrations of inhibitors are separated by gas chromatography and subsequently analyzed by mass-spectrometry, as previously described.[22] The sterol identities were assigned based on relative chromatographic behavior, the characteristic molecular masses and electron ionization (EI) fragmentation patterns of free sterols (**Fig 2**). The sterols were quantified based on the total ion current peak areas of each sterol.

### Catalytic activity of recombinant SMT

The cDNA coding sequence for XP_002680047 was retrieved from NCBI database and the gene was synthesized by Eurofin MWG (Huntsville, AL). The gene was cloned into the PQE30 plasmid (Qiagen) and expressed in *E*. *coli* M15 strain. Briefly, the M15 *E*.*coli* cells harboring expression vector were grown at 37°C in Luria broth (LB) containing ampicillin and kanamycin until OD_600_ reached 0.6. The gene expression was induced by 1.0 mM isopropyl β-D-1-thiogalactopyranoside (IPTG) for 6 hours. The cells were collected by centrifugation and homogenized in 20 mM phosphate buffer in 5% (v/v) glycerol at pH 7.5. After removal of the cell debris by centrifugation at 100,000×g, the soluble fraction was analyzed by Bradford assay to determine the total protein concentration.

To assess the catalytic activity of SMT, thirteen different sterol substrates specified in **Table 2** were used. Enzymatic assays were carried out using crude SMT preparation in 9-ml test tubes containing a total of 600 μl assay volume at 35°C for 4 hours. The assay mixture contained 100 μM sterol emulsified in 12 μl Tween 80 (1.2g/L) and 1.2 mg of total protein. The reaction was initiated by the addition of 200 μM of S-adenosyl methionine. The reactions were terminated with 1 ml of methanolic KOH. Sterols were extracted with hexane (3 x 1 ml) and dried under an N_2_ stream. The extracted sterols were separated by GC/MS and the substrate conversion rate (R) was calculated from the GC peak areas for the substrate (S) and the product (P) peaks according to the following equation: R = 1-P/(S+P). The conversion rates were normalized to the activity of the two best substrates, **5** and **6**, which was considered as 100%.

### Validation of the inhibitors for growth inhibition activity

Compounds were tested against *N*. *fowleri* KUL trophozoites axenically cultured in Nelson’s medium supplemented with 10% fetal bovine serum at 37°C.[22] All the experiments were performed using trophozoites harvested during the logarithmic phase of growth. Screening was performed in triplicate by using an ATP bioluminescence-based assay[40] as previously described.[22] The experiments using trophozoites were conducted in a biosafety cabinet following the BSL2 procedures as specified in the UCSD Biosafety Practices Guidelines.

### Determination of FIC indices and isobologram construction

To determine if the combinations between the inhibitors are synergistic, additive, or antagonistic against *N*. *fowleri* trophozoites, classical isobolograms were used, and combination indices (CI) were calculated as described by Chou and Talalay.[32, 33] Briefly, compounds (epiminolanosterol/tamoxifen, isavuconazole/epiminolanosterol, isavuconazole/tamoxifen) were combined at different micromolar ratios (1:1, 1:2, 1:4, 1:8, 1:16, 2:1, 4:1, 8:1 and 16:1) at each drug concentrations ranging from 50 μM to 0.4 μM (**Fig 4**). Effect for drug pairs was assessed in triplicate in 96-well format with each drug being serially diluted either horizontally or vertically. Growth inhibition was assessed after 48 h using an ATP bioluminescence-based assay.[22]. The results were analyzed using CompuSyn software[32] that calculated combination indices serving as a quantitative measure for drug synergy (CI<1), additivity (CI = 1), or antagonism (CI>1). The dose reduction index (DRI), a fold of dose reduction that allows drug combination to achieve the same degree of inhibition as a dose of the drug used as a single agent, was also calculated by the CompuSyn software.

The predicted synergistic effect of drugs was experimentally tested in a 96-well clear bottom plate. *N*. *fowleri* trophozoites (1×10^4^) were treated with pairs of compounds (isavuconazole/epiminolanosterol, isavuconazole/tamoxifen and epiminolanosterol/tamoxifen) mixed at concentrations that provided the highest CI at 48 h. Cells treated with 0.5% DMSO were used as control. After 48 hours, wells were visually inspected using an Axiovert 40 CFL phase contrast microscope (Carl Zeiss).

## Supporting information

S1 Table Putative sterol biosynthesis genes and ORFs in *Naegleria*. (DOCX)

S2 Table Synergistic effect of drugs. (DOCX)

S1 Text Supporting information references. (DOCX)
