# Supplementary material for: Enzymatic chokepoints and synergistic drug targets in the sterol biosynthesis pathway of Naegleria fowleri
Source: PLoS Pathog. 2018 Sep 13;14(9):e1007245. doi: 10.1371/journal.ppat.1007245 (PMC6136796; doi:10.1371/journal.ppat.1007245)
Supplement: S1 Table — (DOCX) [file ppat.1007245.s001.docx]

**S1 Table.** **Putative sterol biosynthesis genes and ORFs in *Naegleria*.**

| **Enzymes** | ***Gene name^a^/ID*** | | | **Inhibitors** | **% identity** | |
| --- | --- | --- | --- | --- | --- | --- |
|  | ***Yeast*** | ***N. fowleri**** | ***N. gruberi*** |  | ***N. gruberi*** | **Human** |
| **Mevalonate pathway** |  |  |  |  |  |  |
| HMG-CoA reductase | HMG1 | NF0036310 | XP_002670914.1 | Statins[1] | 90% | 56% |
| **Isoprenoid biosynthesis** |  |  |  |  |  |  |
| Farnesyl diphosphate synthase | ERG20 | NF0054070_1 | XP_002682719.1 | Bisphosphonates[2, 3] | 84% | 42% |
| **Sterol biosynthesis** |  |  |  |  |  |  |
| Squalene synthase | ERG9 | NF0115380 | XP_002681803.1 | *S*-thiolofarnesyl diphosphate, quinuclidines, bisphosphonates, thiocyanate | 77% | 41% |
| Squalene epoxidase | ERG1 | NF0065330 | XP_002681586.1  XP_002681587.1 | Allylamines, e.g. terbinafine[4] | 79%  64% | 32% |
| Squalene cyclase | ERG7 | NF0028170 | XP_002682862.1 | Pyridinium-based,[5] dimethylamino truncated squalene ether derivatives[6] | 69% | 39% |
| Cyclopropylsterol isomerase | N/A | NF0065460 | XP_002672776 |  | 83% | absent |
| 14-demethylase, CYP51 | ERG11 | NF0102700 | XP_002681678.1  XP_002678320.1 | Azoles, experimental inhibitors: 4-aminopyridyl, fenarimol, tipifarnib and VNI analogs.[7] | 86%  76% | 37% |
| 14-reductase | ERG24 | NF0003660 | XP_002671837.1  XP_002679915.1 | Morpholines[10, 11] | 81%  71% | 35% |
| 4-methyloxidase | ERG25 | NF0084700  NF0109210  NF0109210 | XP_002683254.1  XP_002670136.1  XP_002683131.1  XP_002668883.1 |  | 64-80% | 35-47% |
| 3-dehydrogenase/  4-decarboxylase | ERG26 | NF0014090 | XP_002679087 |  | 87% | 38% |
| 3-ketoreductase | ERG27 | unknown | unknown |  | - | - |
| 24/28-sterol methyltransferase, SMT | ERG6 | NF0115070 | XP_002680047.1  XP_002671982.1 | Arylguanidines,[8] azasterols[9] | 86%  75% | absent |
| 22-desaturase | ERG5 | unknown | unknown |  | - | N/A |
| Δ24-reductase | ERG4 | NF0103880  NF0103870 | XP_002683543.1  XP_002670768.1 |  | 78-86% | <20%^b^ |
| 5-desaturase | ERG3 | NF0057340 | XP_002683255.1 |  | 78% | 44% |
| Δ^8^-Δ^7^-isomerase | ERG2 | NF0056720 | XP_002682046.1 | Morpholines,[10, 11] tamoxifen, AY9944 | 69% | <20%^c^ |
| Δ7-dehydrocholesterol reductase | N/A | NF0029270 | XP_002674357.1 | AY9944[12] | 86% | 33% |
| 7-desaturase  (DAF-36-like) | N/A | NF0030090 | XP_002682415 |  | 60% | absent |
| *Partially annotated *N. fowleri* genome/transcriptome is available at EUpathDB (<http://eupathdb.org/eupathdb/>). | | | | | | |

^a^ERG nomenclature is according to the convention for *S. cerevisiae.*

^b^Δ24-reduction is performed by the nonhomologous ERG4 in fungi and DHCR24/DWF1 in vertebrates/land plants

^c^Δ^8^-Δ^7^ isomerization is performed by the nonhomologous ERG2 in fungi and EBP/HYD1 in vertebrates/land plants
