# Supplementary material for: Enzymatic chokepoints and synergistic drug targets in the sterol biosynthesis pathway of Naegleria fowleri
Source: PLoS Pathog. 2018 Sep 13;14(9):e1007245. doi: 10.1371/journal.ppat.1007245 (PMC6136796; doi:10.1371/journal.ppat.1007245)
Supplement: S2 Table — (DOCX) [file ppat.1007245.s002.docx]

**S2 Table.** Synergistic effect of drugs.

| **Drug:drug ratio** | **% growth inhibition** | **Combination Index (CI)** | **Dose Reduction Index (DRI)** | | **Dose required to achieved 95% inhibition (µM)** | |
| --- | --- | --- | --- | --- | --- | --- |
| **Epiminolanosterol:tamoxifen** | |  | Epiminolanosterol | Tamoxifen | Epiminolanosterol | Tamoxifen |
| 1:1 | 95 | 0.3±0.1 | 17.8±11 | 4.9±0.9 | 2.9±0.8 | 2.9±0.8 |
| 1:2 | 95 | 0.4±0.1 | 23±13.6 | 3.3±0.8 | 2.2±0.7 | 4.4±1.5 |
| 1:4 | 95 | 0.4±0.1 | 37.4±23.2 | 2.1±0.6 | 1.4±0.4 | 5.4±1.6 |
| 1:8 | 95 | 0.5±0.1 | 58.7±34.4 | 2.1±0.6 | 0.9±0.3 | 6.9±2.4 |
| 1:16 | 95 | 0.6±0.2 | 109.3±71.9 | 1.9±0.5 | 0.5±0.2 | 7.7±2.4 |
| 2:1 | 95 | 0.3±0.1 | 12±7.6 | 6.6±1.2 | 3.8±1.2 | 2.1±0.6 |
| 4:1 | 95 | 0.3±0.2 | 7.9±4.9 | 8.9±2 | 6.4±2 | 1.6±0.5 |
| 8:1 | 95 | 0.4±0.3 | 4.7±2.8 | 10.8±2.9 | 10.8±3.7 | 1.4±0.5 |
| 16:1 | 95 | 0.5±0.4 | 3.4±1.9 | 16.1±5.1 | 14.8±5.7 | 0.9±0.4 |
| **Isavuconazole:epiminolanosterol** | |  | Isavuconazole | Epiminolanosterol | Isavuconazole | Epiminolanosterol |
| 1:1 | 95 | 0.2±0.02 | 12.5±2.4 | 16±3.8 | 0.9±0.1 | 0.9±0.1 |
| 1:2 | 95 | 0.1±0.01 | 21.3±3.02 | 13.7±3.3 | 0.5±0.04 | 1±0.1 |
| 1:4 | 95 | 0.1±0.01 | 47.3±10.9 | 14.9±2.3 | 0.2±0.04 | 1±0.2 |
| 1:8 | 95 | 0.1±0.01 | 102.1±32.2 | 15.8±1.6 | 0.1±0.03 | 0.9±0.2 |
| 1:16 | 95 | 0.1±0.04 | 265.1±91.1 | 23.3±14.2 | 0.1±0.02 | 0.7±0.3 |
| 2:1 | 95 | 0.2±0.01 | 7.6±1 | 19.4±4.4 | 1.5±0.1 | 0.7±0.1 |
| 4:1 | 95 | 0.3±0.01 | 4.9±0.4 | 25.4±6.8 | 2.2±0.1 | 0.6±0.02 |
| 8:1 | 95 | 0.3±0.02 | 3.9±0.2 | 40.9±12.2 | 2.8±0.1 | 0.4±0.01 |
| 16:1 | 95 | 0.2±0.01 | 6.3±0.2 | 132.2±37.7 | 1.7±0.1 | 0.1±0.01 |
| **Isavuconazole:tamoxifen** | |  | Isavuconazole | Tamoxifen | Isavuconazole | Tamoxifen |
| 1:1 | 95 | 0.4±0.02 | 5.5±0.4 | 5.7±0.3 | 2±0.1 | 2±0.1 |
| 1:2 | 95 | 0.4±0.03 | 6.6±0.2 | 3.4±0.3 | 1.6±0.1 | 3.3±0.3 |
| 1:4 | 95 | 0.5±0.1 | 9.3±0.6 | 2.5±0.2 | 1.2±0.1 | 4.7±0.4 |
| 1:8 | 95 | 0.7±0.03 | 12.4±0.6 | 1.6±0.1 | 0.9±0.04 | 7.1±0.4 |
| 1:16 | 95 | 0.9±0.1 | 19.2±1.4 | 1.2±0.1 | 0.6±0.04 | 9.1±0.7 |
| 2:1 | 95 | 0.4±0.02 | 3.8±0.3 | 7.9±0.6 | 2.9±0.2 | 1.5±0.1 |
| 4:1 | 95 | 0.4±0.03 | 3±0.2 | 12.4±0.5 | 3.7±0.03 | 0.9±0.01 |
| 8:1 | 95 | 0.5±0.04 | 2.3±0.2 | 19.5±2.5 | 4.7±0.6 | 0.6±0.1 |
| 16:1 | 95 | 0.6±0.04 | 1.9±0.1 | 31±3.0 | 5.9±0.5 | 0.4±0.03 |
