# Supplementary material for: Enzymatic chokepoints and synergistic drug targets in the sterol biosynthesis pathway of Naegleria fowleri
Source: PLoS Pathog. 2018 Sep 13;14(9):e1007245. doi: 10.1371/journal.ppat.1007245 (PMC6136796; doi:10.1371/journal.ppat.1007245)
Supplement: S1 Text — (DOCX) [file ppat.1007245.s003.docx]

**S1 Text. References for S1 and S2 Tables.**

1. Urbina JA, Lazardi K, Marchan E, Visbal G, Aguirre T, Piras MM, et al. Mevinolin (lovastatin) potentiates the antiproliferative effects of ketoconazole and terbinafine against *Trypanosoma (Schizotrypanum) cruzi*: *in vitro* and *in vivo* studies. Antimicrob Agents Chemother. 1993;37(3):580-91.

2. Martin MB, Grimley JS, Lewis JC, Heath HT, 3rd, Bailey BN, Kendrick H, et al. Bisphosphonates inhibit the growth of *Trypanosoma brucei, Trypanosoma cruzi, Leishmania donovani, Toxoplasma gondii*, and *Plasmodium falciparum*: a potential route to chemotherapy. J Med Chem. 2001;44(6):909-16.

3. Docampo R, Moreno SN. Bisphosphonates as chemotherapeutic agents against trypanosomatid and apicomplexan parasites. Curr. Drug Targets Infect Disord. 2001;1(1):51-61.

4. Vannier-Santos MA, Urbina JA, Martiny A, Neves A, de Souza W. Alterations induced by the antifungal compounds ketoconazole and terbinafine in *Leishmania*. J Eukaryot Microbiol. 1995;42(4):337-46.

5. Buckner FS, Griffin JH, Wilson AJ, Van Voorhis WC. Potent anti-*Trypanosoma cruzi* activities of oxidosqualene cyclase inhibitors. Antimicrob Agents Chemother. 2001;45(4):1210-5.

6. Oliaro-Bosso S, Ceruti M, Balliano G, Milla P, Rocco F, Viola F. Analogs of squalene and oxidosqualene inhibit oxidosqualene cyclase of *Trypanosoma cruzi* expressed in *Saccharomyces cerevisiae*. Lipids. 2005;40(12):1257-62.

7. Choi JY, Podust LM, Roush WR. Drug strategies targeting CYP51 in neglected tropical diseases. Chem Rev. 2014;114(22):11242-71.

8. Borelli C, Schaller M, Niewerth M, Nocker K, Baasner B, Berg D, et al. Modes of action of the new arylguanidine abafungin beyond interference with ergosterol biosynthesis and in vitro activity against medically important fungi. Chemotherapy. 2008;54(4):245-59.

9. Gros L, Lorente SO, Jimenez CJ, Yardley V, Rattray L, Wharton H, et al. Evaluation of azasterols as anti-parasitics. J Med Chem. 2006;49(20):6094-103.

10. Mercer EI. Morpholine antifungals and their mode of action. Biochem Soc Trans. 1991;19(3):788-93.

11. Moebius FF, Bermoser K, Reiter RJ, Hanner M, Glossmann H. Yeast sterol C8-C7 isomerase: identification and characterization of a high-affinity binding site for enzyme inhibitors. Biochemistry. 1996;35(51):16871-8.

12. Kolf-Clauw M, Chevy F, Wolf C, Siliart B, Citadelle D, Roux C. Inhibition of 7-dehydrocholesterol reductase by the teratogen AY9944: a rat model for Smith-Lemli-Opitz syndrome. Teratology. 1996;54(3):115-25.
